# Female Dynamics in Authorship of Scientific Publications in the Public Library of Science: A 10-year Bibliometric Analysis of Biomedical Research

**DOI:** 10.3390/ejihpe13020018

**Published:** 2023-01-20

**Authors:** Panagiotis Giannos, Konstantinos Katsikas Triantafyllidis, Maria Paraskevaidi, Maria Kyrgiou, Konstantinos S. Kechagias

**Affiliations:** 1 Society of Meta-Research and Biomedical Innovation, London W12 0FD, UK; 2 Department of Life Sciences, Faculty of Natural Sciences, Imperial College London, London SW7 2BX, UK; 3 Department of Nutrition & Dietetics, Musgrove Park Hospital, Taunton TA1 5DA, UK; 4 Department of Metabolism, Digestion and Reproduction, Faculty of Medicine, Imperial College London, London SW7 2BX, UK; 5 Institute of Reproductive and Developmental Biology, Imperial College London, London SW7 2BX, UK; 6 West London Gynaecological Cancer Centre, Imperial College NHS Trust, London W6 8RF, UK

**Keywords:** gender, equality, diversity, research, PLoS

## Abstract

Women are generally underrepresented in science, technology, engineering, and mathematics (STEM). As scientific production reflects scholarly impact and participation in the scientific process, the number of journal publications forms a pertinent measure of academic productivity. This study examined the prevalence and evolution of female representation in prominent author positions across multidisciplinary biomedical research. Publications from seven exemplar cross-specialty journals of the Public Library of Science (*PLoS Medicine, PLoS Biology, PLoS One, PLoS Computational Biology, PLoS Genetics, PLoS Pathogens, and PLoS Neglected Tropical Diseases*) between January 2010 and December 2020 were extracted from Web of Science. Using Genderize.io, the gender of authors from their first names was estimated using a 75% threshold. The association between female prevalence in first and last authorship and journal was evaluated using a binary logistic regression, and odds ratios were estimated against a 50:50 reference on gender. In 266,739 publications, 43.3% of first authors and 26.7% of last authors were females. Across the ten-year period, female first authorship increased by 19.6% and last authorship by 3.2%. Among all journals, *PLoS Neglected Tropical Diseases* had the greatest total proportion of female first authors (45.7%) and *PLoS Medicine* of female last authors (32%), while *PLoS Computational Biology* had the lowest proportion in these categories (23.7% and 17.2%). First authors were less likely to be females in all PLoS journals (*p* < 0.05) except for *PLoS Neglected Tropical Diseases* (odds ratio: 0.84, 95% confidence interval: 0.71–1.00), where the odds of female authorship were not significantly different (*p* = 0.054). Last authors were not more likely to be females in all PLoS journals (*p* < 0.001). Overall, women still appear underrepresented as first authors in biomedical publications and their representation as last authors has severely lagged. Efforts towards gender equality in scholarly authorship will contribute to the representation of women in biomedical research and ensure that their potential is not lost.

## 1. Introduction

The proportion of women who are engaged academically and professionally across the fields of science, technology, engineering, and mathematics (STEM) has been increasing in recent decades [1]. However, the underrepresentation of women in leading academic positions still persists. The increasing number of degrees awarded to female graduates at both master’s and doctoral levels has been well documented [2], however, women’s representation in academia stills lags behind, especially among senior faculty. One study in the United Kingdom has highlighted that female staff accounted for 46% among those on an academic contract, with those listed as professors at 27% and other senior positions at 38% [3]. The unequal distribution between genders has further extended to the underrepresentation of women in academic research. Female scientists have often less available resources to them, receive lower research funding than their male colleagues, and are overall less likely to be listed as principal investigators [4,5,6,7,8,9].

There has also been considerable gender inequality in the authorship of scientific production and throughout the publication process itself. Evidence from a series of studies, have highlighted a significant underrepresentation of women in prominent author positions [10,11,12,13,14]. Most prominently, a worldwide review of research output from six highly ranked medical journals revealed that women comprise a minority in relation to first and last authors [10]. In fact, publications with female first and last authors attracted fewer citations than those with males in the same roles [15]. This slow participation of women in the scientific process has been further exemplified by the lower gender diversity on journal editorial boards [16,17,18,19,20,21] and scientific committees [22,23].

A plethora of studies have already attempted to describe the overall publication trends of female authors across STEM but without addressing this in areas of interdisciplinary biomedical research [24]. A majority has also placed emphasis on subscription-based high-impact factor journals, which are classically perceived to be of increased visibility. However, impact factor does not necessarily echo the wider perception of researchers about the journals’ content, and its high score often reflects a large number of citations linked to a small number of publications [25,26,27,28,29,30,31,32,33]. In fact, editors in highly ranked journals often prioritise publications that will likely attract more citations [34]. Nevertheless, publishing in subscription-based journals does not require (most often expensive) article processing-charges as opposed to those being open access (OA). This in turn does not create an opportunity to reflect the contribution of grant-funded researchers and their merit beyond representation through authorship. A previous report has suggested that medical researchers could have had a cost burden up to USD $34,676 for publishing due to article processing charges [35]. Hence, as more journals become OA, assessing the research productivity and funded inclusion of females in biomedical research becomes crucial.

Advancement within the academic ladder is largely contingent on scientific production and scholarly impact. Widely available metrics pertained to academic productivity and scientific influence include the publication number of peer-reviewed research. In our study, we aimed to examine the prevalence and evolution of female representation in prominent author roles—first and last authorship—and determine whether quantifiable gender differences across multidisciplinary biomedical research, exist. To accomplish the purpose of our study, we focused as an example on publications from seven cross-specialty OA journals of the Public Library of Science (PLoS) between 2010 and 2020.

## 2. Materials and Methods

We examined the prevalence of female first and last authors in publications from seven PLoS journals between 1 January 2010 and 31 December 2020. Initially, we selected these as they offer a representation of an exemplar sample of multidisciplinary biomedical research because of their cross-speciality series of journals. Thereafter, as the journals operate on OA and thus, article processing charges apply to publishing, authorship trends observed here may reflect the contribution of grant-funded researchers and their inclusion. Lastly, focusing on a single publisher allows for horizontal comparisons to be made more appropriately since different publishers may have variable priorities for publishing different or specific types of studies.

We performed a literature search using the Web of Science (WoS) database (http://scientific.thomson.com/products/wos/ (accessed on 5 February 2021) to retrieve full bibliographic records of all peer-reviewed publications from each year in *PLoS Medicine*, *PLoS Biology*, *PLoS One*, *PLoS Computational Biology*, *PLoS Genetics*, *PLoS Pathogens*, and *PLoS Neglected Tropical Diseases*. All types of publications were considered in our study and hence, our search on WoS was only limited to the aforementioned journal titles as search terms. No exclusion criteria were applied in our search strategy to avoid selection bias.

Extracted publications were grouped by year and journal and the full name of the authors, article title, and digital object identifier of these publications were retrieved. Considering that authorship name was exported by the WoS database as a continuous string of all listed authors, all name entries were processed locally and the full names of the first and last authors were retrieved using a set of character-based delimiters. Publications without any named authors and with single-named authors or those listed with their initials were omitted.

The gender of the first and last authors was predicted using the Genderize.io database (https://genderize.io (accessed on 12 February 2021); Demografix ApS, Roskilde, Denmark) by determining the likelihood of association between their first names and a binary gender category. This gender prediction method utilises known genders collected from user profiles on major social networks. To assign names with a specific gender (classified as “Male” or “Female”), we used a 75% probability threshold. Authors whose gender could not be determined based on their first names (classified as “Unknown”) were excluded from all further analyses.

We performed a binary logistic regression analysis to detect the potential association between female prevalence in first and last authorship and journal, using the statistical software SPSS 26 (IBM Corp., Armonk, NY, USA). Statistical significance was established as *p* < 0.05. In our model, we treated gender as a dichotomous dependent variable, while journal was used separately as an independent variable. Using the same model, we estimated the odds ratio of a publication having a female first and last author for all included journals. A 50:50 mean of female first and last authors across the seven journals and over the ten-year period was used as the reference groups.

## 3. Results

Our initial search yielded 266,739 publications from seven cross-specialty PLoS journals, between 2010 and 2020. Among these, 262,122 publications were eligible for first-author gender prediction, with the remaining 4617 (1.8%) publications being omitted (non-listed authors (N = 292), single-named authors (N = 2), and authors with initials (N = 4323)). Similarly, 261,799 publications were suitable for last-author gender prediction and 4940 (1.9%) publications were excluded (non-listed authors (N = 292), single-named authors (N = 190), and authors with initials (N = 4458)). Ultimately, we were able to predict the gender of 211,404 (79.3%) first authors and 221,794 (86.1%) last authors. Of the first authors, 91,619 (43.3%) were female, while of the last authors, 59,208 (26.7%) were female.

**Figure 1:**
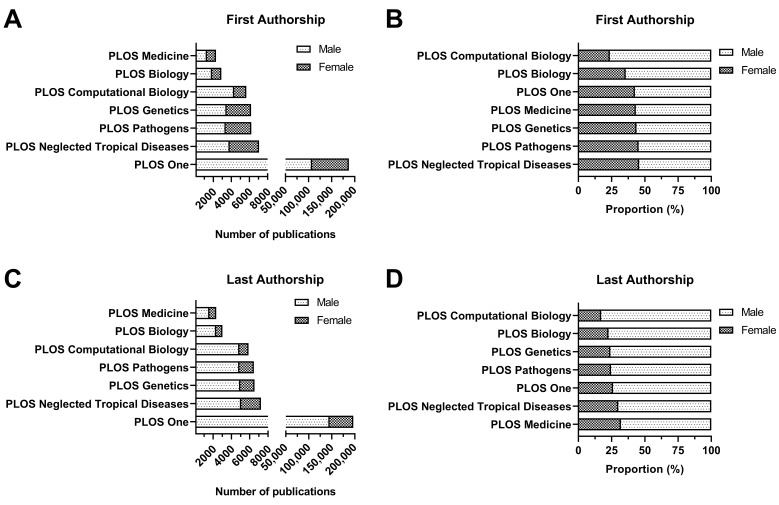
Total count and proportion of first authors (**A**,**B**) and last authors (**C**,**D**) by gender among publications from seven cross-specialty journals of the Public Library of Science (PLoS) between 2010 and 2020.

**Figure 2:**
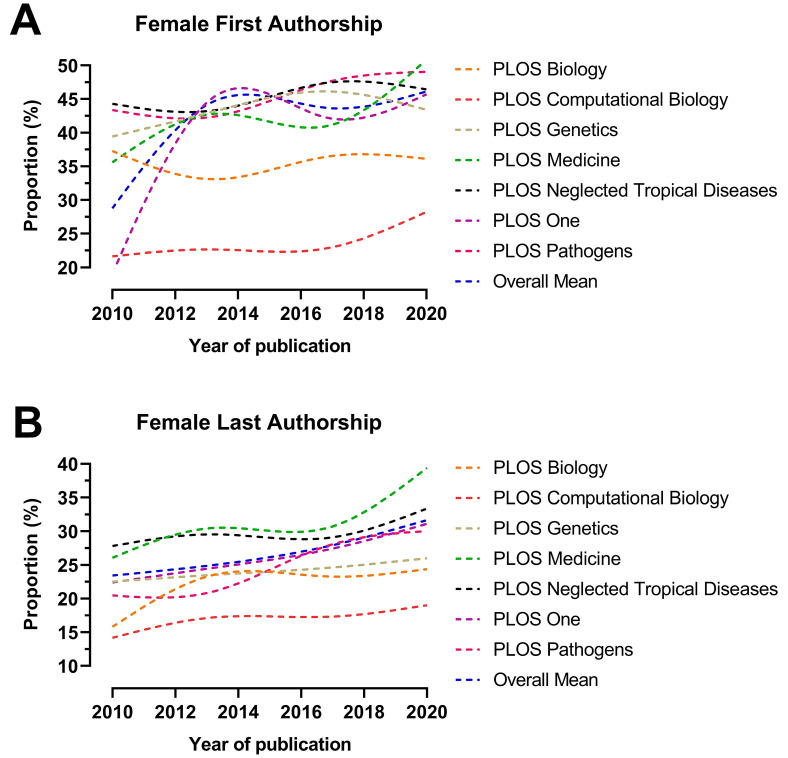
Temporal trends of female authorship—first authorship (**A**) and last authorship (**B**)—in publications from seven cross-specialty journals of the Public Library of Science (PLoS) between 2010 and 2020. Annual female prevalence was modelled using restricted cubic splines.

The total number and proportion of female first and last authors in publications published each year and across the ten-year period in the included journals are summarised in Table S1. *PLoS Neglected Tropical Diseases* had the greatest total proportion of female first authors (3223/7057; 45.7%) and *PLoS Medicine* of female last authors (745/2331; 32%) over the ten-year period, while *PLoS Computational Biology* had the lowest proportion in these categories (1338/5653, 23.7% and 1011/5876, 17.2%) (Figure 1). Further, *PLoS Medicine* had the highest proportion of female first authors (158/296; 53.4%) and female last authors (121/307; 39.4%) in 2020, while *PLoS Computational Biology* had the lowest proportion in these categories (193/679; 28.4% and 139/711; 19.5%) (Figure 2).

The overall proportion of female first and last authors increased across the majority of journals between 2010 and 2020. Particularly, female first authorship increased from 23.7% in 2010 to 44.9% in 2020, followed by an increase in female last authorship from 23.5% in 2010 to 31.4% in 2020. The highest increase in female first authorship was in *PLoS One* (32.9%) and in female last authorship in *PLoS Medicine* (14.1%). The lowest change in female first authorship was in *PLoS Biology* (−1.9%) and in female last authorship in *PLoS Genetics* (2.3%) (Figure 2).

**Figure 3:**
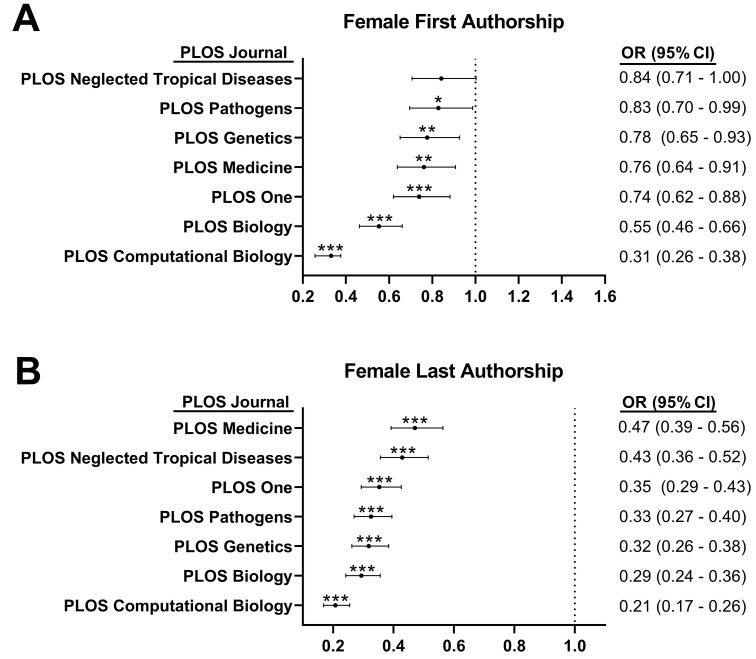
Odds ratio (OR) and 95% confidence intervals (CI) of female authorship—first authorship (**A**) and last authorship (**B**)—in publications from seven cross-specialty journals of the Public Library of Science (PLoS) between 2010 and 2020. A 50:50 mean of female first and last authors across the seven journals and over the ten-year period was used as the reference groups. * *p* < 0.05, ** *p* < 0.01, *** *p* < 0.001.

The female-to-male odds ratio analysis showed that females had statistically significant lower odds for first authorship in all PLoS journals [*PLoS Pathogens* (odds ratio (OR): 0.83, 95% confidence interval (CI): 0.70–0.99, *p* = 0.035), *PLoS Genetics* (OR: 0.78, 95% CI: 0.65–0.93, *p* = 0.005), *PLoS Medicine* (OR: 0.76, 95% CI: 0.64–0.91, *p* = 0.002), PLoS One (OR: 0.74, 95% CI: 0.62–0.88, *p* < 0.001), *PLoS Biology* (OR: 0.55, 95% CI: 0.46–0.66, *p* < 0.001), and *PLoS Computational Biology* (OR: 0.31, 95% CI: 0.26–0.38, *p* < 0.001)], except for *PLoS Neglected Tropical Diseases* (OR: 0.84, 95% CI: 0.71–1, *p* = 0.054), where the odds of female authorship were not significantly different. By contrast, females had statistically significant lower odds for last authorship in all PLoS journals [*PLoS Medicine* (OR: 0.47, 95% CI: 0.39–0.56, *p* < 0.001), *PLoS Neglected Tropical Diseases* (OR: 0.43, 95% CI: 0.36–0.52, *p* < 0.001), *PLoS One* (OR: 0.35, 95% CI: 0.29–0.43, *p* < 0.001), *PLoS Pathogens* (OR: 0.33, 95% CI: 0.27–0.4, *p* < 0.001), *PLoS Genetics* (OR: 0.32, 95% CI: 0.26–0.38, *p* < 0.001), *PLoS Biology* (OR: 0.29, 95% CI: 0.24–0.36, *p* < 0.001), and *PLoS Computational Biology* (OR: 0.21, 95% CI: 0.17–0.26, *p* < 0.001)] (Figure 3).

## 4. Discussion

Our findings highlighted a gender gap in first and last authorship across publications from seven OA cross-specialty PLoS journals in biomedical research, between 2010 and 2020. We demonstrated that less than a half of the first authors and almost a fourth of the last authors, were females. Likewise, female first authorship increased by 21.2% and last authorship by 7.9% across the ten-year period. Indeed, first and last authors were less likely to be females in the majority of PLoS journals. *PLoS Computational Biology* had the lowest total proportion in female first and last authorship over the ten-year period and in 2020, with overall less than a fourth of first authors and almost a fifth of last authors, as females. Nevertheless, our results showed a slow narrowing of the gender gap for first authors and last author positions. Indicative of this, *PLoS Neglected Tropical Diseases* had the greatest total proportion among first authors with almost a half as females, and *PLoS Medicine* among last authors with more than a third being females. Equally, female firth authorship increased highest, by a third, in *PLoS One* and female last authorship by less than a fifth in *PLoS Medicine*. 

We have identified a continuous increase in the representation of women among first and last authors across most PLoS journals between 2010 and 2020. Although our data did not allow us to unveil the underlying mechanism of this change, our results consistently demonstrated that a gender gap in scientific authorship still exists, especially among last authors. While there are no prior studies examining gender authorship in PLoS journals, our findings are in line with previous reports that focused on different but relevant biomedical journals, pointing out an increasing trend in the prevalence of female first authorship, but with that, last authorship is yet to catch up. Specifically, a study which examined the gender distribution across the biosciences and within the entire JSTOR corpus highlighted a more pronounced decrease, specifically in the number of female last authors as opposed to that in first authors which was less severe [14].

Multiple inter-connected explanations could likely underlie our observation. Firstly, there may be a “leaky pipeline” with each step up in the academic ladder, a phenomenon which has long been described and acknowledged by the scientific literature [36,37,38]. During the transition from being a graduate student to an early faculty member, and before progressing to a senior member, a decrease in the number of women has been observed in academic research, when compared to men [36,37,38,39]. Secondly, evidence has demonstrated the role of implicit bias in preventing women from being promoted to more senior roles by creating an academic environment that does not adequately support them, especially during child rearing [40,41,42,43]. Particularly, a large percentage of postdoctoral women have reported that they would not consider pursuing a principal investigator position [39,44,45,46]. Additionally, reduced career confidence and lower propensity to apply for faculty positions have also been observed among women [44,47,48]. Lastly, the shortage of women in senior positions within academia may simply be a result of a “demographic inertia”, which suggests that because women scholars were fewer in the past, there may be a lag period of time before women reach more senior positions [49]. In all cases, deciphering whether the lower representation of women among authors is due to their exclusion from authorship or from the research process itself remains essential, considering that female representation among authors could be increased without merit. Future self-initiatives by other OA publishers similar to PLoS, are essential to inform whether publications practices acknowledge equal scholarly representation in biomedical research from a gender perceptive.

Our results also demonstrated that two prominent PLoS journals—*PLoS Neglected Tropical Diseases* and *PLoS Computational Biology*—have greatly opposing patterns in female representation across first and last authorship. To date, there are no previous studies looking at the prevalence of gender authorship in the field of neglected tropical diseases across the biomedical literature. However, reports have highlighted an underrepresentation of women in infectious diseases research. In a review examining gender parity in *The Lancet Infectious Diseases*, females constituted 32% of first authors and 22% of last authors between 2014 and 2017 [50]. However, another line of evidence suggested that 42% of active clinicians in the respective specialty in 2019 were women, a percentage higher than the average across all specialties (32%) [51]. Nevertheless in 2014, significant disparities were observed in female clinicians of this specialty when considering publication productivity and the potential of achieving full professor rank [52]. Thus, investigating the gender distribution in female authorship specifically within the field of neglected tropical diseases is essential in shedding light against the scarcity of current evidence.

Moreover, there is ample literature on the unequal distribution between genders in STEM across all aspects of scientific and academic practice. Yet, there are only a few studies examining gender authorship in interdisciplinary fields such as computational biology and a lot more about its parent fields of biology and computer science. When looking in doctoral degrees awarded to women, computer science has the lowest share of recipients, while in biology, more than a half of degrees are held by women [53]. Within this spectrum, a study demonstrated that computational biology lies in between these two fields [54], with less than a third of first authors and almost a fifth of last authors being women across publications from 1997 to 2014. In this way, although computational biology may appear a more conducive environment to female participation, significant gender differences within this interdisciplinary field still exist and warrant interventions designed to help narrowing the present gap.

Our study provided interesting insights about female dynamics in authorship among PLoS journals in biomedical research, yet methodological and conceptual limitations exist. Primarily, we inferred gender of authorship using the Genderize.io database which is based on user profiles from social networks, that may contain arbitrary words or characters which could have contributed to a less accurate gender estimation [55]. Similarly, there is also evidence that this tool has limited confidence in predicting gender names of Asian origin [55]. In fact, its prediction of gender is limited to a binary category, without accounting for self-identified gender and non-binary or gender-neutral names. Thus, suggesting the potential difficulties of studying gender dichotomously. However, this is a prominent limitation across the majority of gender inference services, which suggests an unmet need of accounting gender beyond female and male categories. Nevertheless, previous reports have demonstrated a substantially lower misclassification rate on gender assignment when using the Genderize.io database over other gender inference services [55]. We also focused on OA journals that are prone to expensive article processing-charges, which may limit the ability for non-grant-funded researchers to publish in these journals. However, the underrepresentation of women in these journals could reflect the true state of grant funding to female researchers and the discordance of resources offered to them and their male colleagues [56,57]. Further, our analysis did not account for dual first author publications and any factors other than the year published. Interestingly, the proportion of female first authors has been shown to be variable between experimental and non-experimental studies [58], while in the field of computational biology, women are significantly less likely to be authors if the publication did not include a female last author [54]. Additionally, other parameters such as geographic origin under which the included studies were conducted, has shown to exhibit an interacting effect on female authorship [15]. Lastly, PLoS has been a long advocate of diversity, equity, and inclusion in terms of scholarly participation. Yet, it remains unclear as to whether editorial processes in the form of publication policies were indeed employed across the former time duration, to encourage female authorship. In this sense, it is probable that countries which chose to publish in PLoS may have egalitarian research policies, that may have spill-over effects in the form of research bias and publication. 

## 5. Conclusions

Our study showed that women are underrepresented in seven cross-specialty PLoS journals and confirms previous studies done in other journal sets and disciplines. Although our findings highlighted a slow narrowing of an existing gap among female first authors, women’s representation as last authors has severely lagged behind. This reinforces the potential attrition of women in academic career advancement with probable barriers at the levels of research support, dedicated research time, or the publication process itself. Our study reinforced that we cannot yet disregard disparities in female authorship and hint that this matter warrants renewed attention. While proposing interventions towards gender equality was beyond the focus of our study, we should emphasise their importance in maximising the contribution of women in research and ensuring that their potential is not lost.

## Data Availability

Publicly available data were analysed in this study, and these can be found in the Web of Science database.

## Supplementary Materials

The following supporting information can be downloaded at: https://www.mdpi.com/article/10.3390/ejihpe13020018/s1, Table S1. Representation of female first and last authorship in publications from seven cross-specialty journals of the Public Library of Science between 2010 and 2020. Values are presented as numbers (percentages).
