# Supplementary material for: Female Dynamics in Authorship of Scientific Publications in the Public Library of Science: A 10-year Bibliometric Analysis of Biomedical Research
Source: Eur J Investig Health Psychol Educ. 2023 Jan 20;13(2):228–37. doi: 10.3390/ejihpe13020018 (PMC9955408; doi:10.3390/ejihpe13020018)
Supplement: Supplementary file 1 [file ejihpe-13-00018-s001.zip › ejihpe-2051038-supplementary.pdf]

Supplementary Tables

Table S1. Representation of female first and last authorship in publications from seven cross-specialty journals of the Public Library of Science between 2010 and 2020. Values are presented as numbers (percentages).

	2010	2011	2012	2013	2014	2015	2016	2017	2018	2019	2020	Total
**First Authorship**												
**Biology**	83/221 (37.6)	69/197 (35)	57/171 (33.3)	90/263 (34.2)	82/247 (33.2)	85/249 (34.1)	86/243 (35.4)	81/217 (37.3)	127/355 (35.8)	159/423 (37.6)	111/311 (35.7)	1030/2897 (35.6)
**Computational Biology**	77/374 (20.6)	91/374 (24.3)	100/467 (21.4)	112/501 (22.4)	121/515 (23.5)	119/574 (20.7)	121/520 (23.3)	126/535 (23.6)	122/517 (23.6)	156/597 (26.1)	193/679 (28.4)	1338/5653 (23.7)
**Genetics**	169/421 (40.1)	194/502 (38.6)	279/638 (43.7)	325/767 (42.4)	297/690 (43)	314/668 (47)	250/565 (44.2)	229/483 (47.4)	229/509 (45)	214/478 (44.8)	199/459 (43.4)	2699/6180 (43.7)
**Medicine**	65/180 (36.1)	70/189 (37)	78/189 (41.3)	92/199 (46.2)	77/178 (43.3)	43/128 (33.6)	101/228 (44.3)	117/250 (46.8)	84/208 (40.4)	108/251 (43)	158/296 (53.4)	993/2296 (43.2)
**Neglected Tropical Diseases**	141/310 (45.5)	165/410 (40.2)	219/477 (45.9)	242/557 (43.4)	302/700 (43.1)	333/739 (45.1)	369/796 (46.4)	393/804 (48.9)	325/699 (46.5)	338/711 (47.5)	396/854 (46.4)	3223/7057 (45.7)
**One**	311/2942 (10.6)	4783/11783 (40.6)	8179/19603 (41.7)	10963/25800 (42.5)	10415/24589 (42.4)	10101/23653 (42.7)	8132/18679 (43.5)	7662/17313 (44.3)	6955/15798 (44)	6105/13539 (45.1)	5921/13622 (43.5)	79527/187321 (42.5)
**Pathogens**	210/493 (42.6)	208/489 (42.5)	256/584 (43.8)	284/654 (43.4)	255/596 (42.8)	254/623 (40.8)	284/609 (46.6)	278/543 (51.2)	250/517 (48.4)	222/475 (46.7)	308/617 (49.9)	2809/6200 (45.3)
**All**	1056/4448 (23.7)	5580/13455 (41.5)	9168/21545 (42.6)	12108/28087 (43.1)	11549/26919 (42.9)	11249/26011 (43.2)	9343/21031 (44.4)	8886/19602 (45.3)	8092/18086 (44.7)	7302/15999 (45.6)	7286/16221 (44.9)	91619/211404 (43.3)
**Last Authorship**												
**Biology**	43/231 (18.6)	29/206 (14.1)	41/187 (21.9)	65/271 (24)	64/255 (25.1)	67/261 (25.7)	49/246 (19.9)	57/234 (24.4)	91/369 (24.7)	98/433 (22.6)	80/324 (24.7)	684/3017 (22.7)
**Computational Biology**	54/394 (13.7)	62/390 (15.9)	83/484 (17.1)	82/521 (15.7)	103/541 (19)	92/599 (15.4)	100/539 (18.6)	90/546 (16.5)	98/512 (19.1)	108/639 (16.9)	139/711 (19.5)	1011/5876 (17.2)
**Genetics**	103/443 (23.3)	115/526 (21.9)	149/666 (22.4)	197/802 (24.6)	170/738 (23)	186/716 (26)	135/607 (22.2)	129/517 (25)	134/534 (25.1)	129/494 (26.1)	125/488 (25.6)	1572/6531 (24.1)
**Medicine**	45/178 (25.3)	58/199 (29.1)	56/189 (29.6)	60/198 (30.3)	52/172 (30.2)	38/133 (28.6)	72/232 (31)	86/252 (34.1)	58/209 (27.8)	99/262 (37.8)	121/307 (39.4)	745/2331 (32)

**Neglected Tropical Diseases**	95/332 (28.6)	110/415 (26.5)	149/480 (31)	171/574 (29.8)	200/718 (27.9)	225/769 (29.3)	240/816 (29.4)	259/829 (31.2)	205/733 (28)	210/694 (30.3)	303/874 (34.7)	2167/7234 (30)
**One**	1371/6138 (22.3)	2802/12226 (22.9)	4970/20341 (24.4)	6488/26725 (24.3)	6333/25464 (24.9)	6251/24389 (25.6)	5147/19370 (26.6)	4849/17936 (27)	4693/16306 (28.8)	4252/13919 (30.5)	4285/13991 (30.6)	51441/196805 (26.1)
**Pathogens**	105/511 (20.5)	103/516 (20)	122/585 (20.9)	132/687 (19.2)	153/633 (24.2)	163/651 (25)	145/623 (23.3)	160/568 (28.2)	162/536 (30.2)	159/505 (31.5)	184/645 (28.5)	1588/6460 (24.6)
**All**	1816/7716 (23.5)	3279/13962 (23.5)	5570/22347 (24.9)	7195/29091 (24.7)	7075/27888 (25.4)	7022/26867 (26.1)	5888/21810 (27)	5630/20314 (27.7)	5441/18663 (29.2)	5055/16441 (30.7)	5237/16695 (31.4)	59208/221794 (26.7)
